# Supplementary material for: Amorphous Solid Dispersions and the Contribution of Nanoparticles to In Vitro Dissolution and In Vivo Testing: Niclosamide as a Case Study
Source: Pharmaceutics. 2021 Jan 14;13(1):97. doi: 10.3390/pharmaceutics13010097 (PMC7828663; doi:10.3390/pharmaceutics13010097)
Supplement: Supplementary file 1 [file pharmaceutics-13-00097-s001.pdf]

pharmaceutics

Supplementary Materials: Amorphous Solid Dispersions and the Confounding Effect of Nanoparticles in In Vitro Dissolution and In Vivo Testing: Niclosamide as a Case Study

Miguel O. Jara, Zachary N. Warnken and Robert O. Williams III

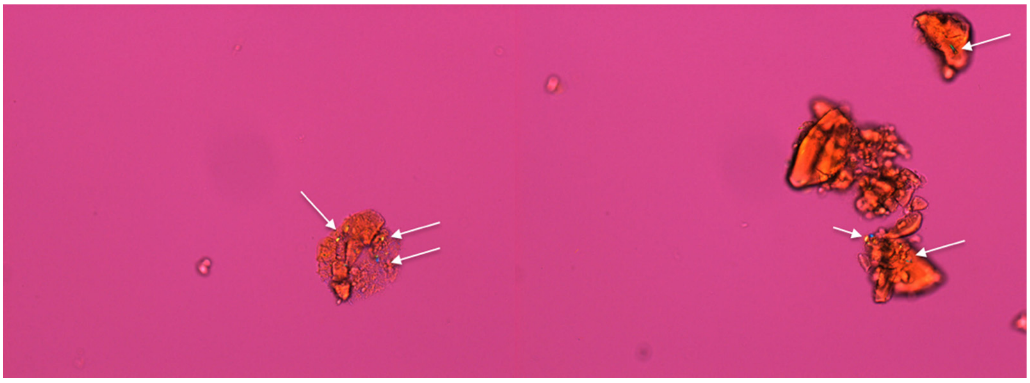

Figure S1. PLM of niclosamide ASD at 24 h without pH-shift. Signs of crystallization were observed (white arrows).

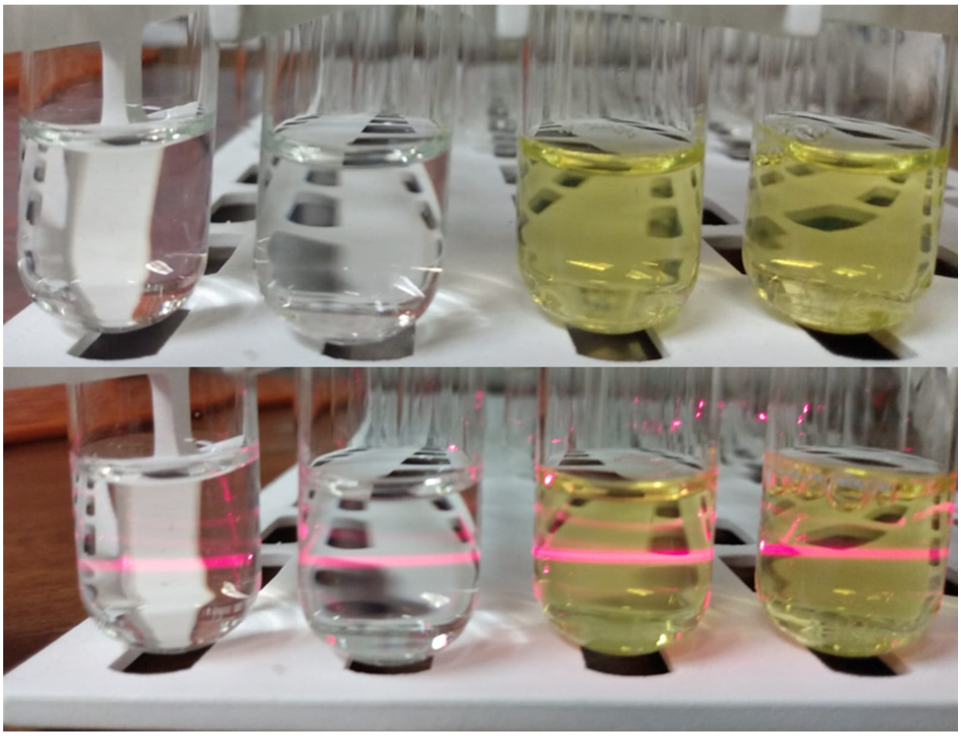

Figure S2. The image shows the appearance of samples for HPLC after filtration. The samples in the right are with pH-shift (transparent) and in the left without pH-shift after 24 h. The laser beam shows the presence of colloidal species. The yellow color is related to higher concentrations of niclosamide.

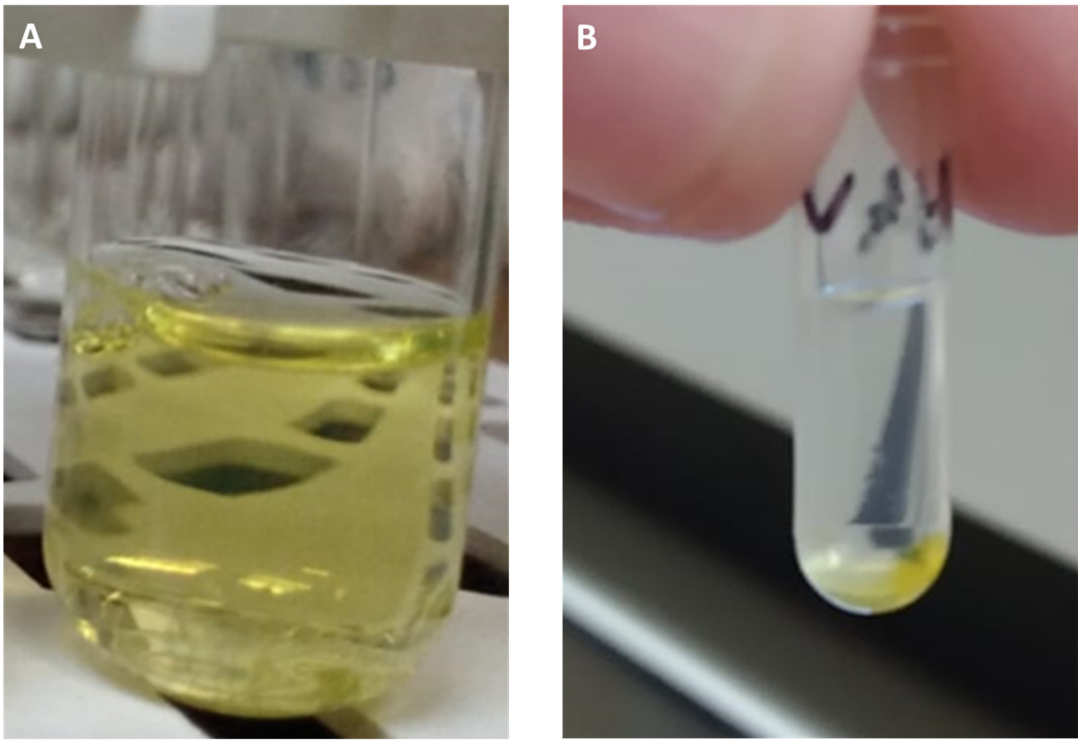

Figure S3. (**A**) Shows a sample directly taken from the dissolution vessel after passing through the 0.2 µm filter (420 µg/mL). (**B**) Shows a sample like Figure A after undergoing ultracentrifugation (11 µg/mL).

Table S1. Mobile phase gradient that was used for analyzing plasma sample.

Time (min)	A (%)	B (%)
0.20	85.0	15.0
2.00	50.0	50.0
2.50	50.0	50.0
4.00	0.00	100
4.50	0.00	100
4.51	85.0	15.0
5.00	85.0	15.0

Table S2. Mean particle size, PDI, and zeta potential of supernatants after centrifugation at 13,000 rpm x 10 min. The samples were taken from the dissolution apparatus at different time points. It can be noted that FaSSIF helps in the generation of smaller nanoparticles.

Sample	Sampling Time (h)	Mean Particle Size (d nm)	PDI	Zeta Potential (mV)
FaSSIF Media	1	66.5 ± 0.09	0.037 ± 0.048	−14.8 ± 2.3
Niclosamide ASD in Buffer 6.5	1	228.1 ± 4.2	0.157 ± 0.011	−12.1 ± 0.1
Niclosamide ASD in FaSSIF	1	99.3 ± 1.4	0.224 ± 0.004	−13.6 ± 1.0
